# Fake lifejackets play a role in drowning of refugees

**DOI:** 10.2471/BLT.16.020616

**Published:** 2016-06-01

## Abstract

Fake lifejackets are one of the hazards refugees face when they risk their lives to cross the sea to Europe. Menelaos Tzafalias reports from the Greek island of Lesbos.

Last September, Nikolaos Andreou saw a blue object on the seafloor in front of his house on the Greek island of Lesbos. The object was a brand-new-looking lifejacket.

“I hoisted it out of the sea to let it dry, but when I picked it up it was very heavy. That puzzled me because a lifejacket is supposed to float,” he says.

Andreou is a marine engineer who worked for several years as a technical inspector for a passenger shipping company. His responsibilities included purchasing safety equipment and authorizing the equipment’s use in accordance with EU regulations.

“I hung up the lifejacket on my clothes line. Later that afternoon I tested it, attaching a 10-kg weight to it and putting it back in the water. It was an adult lifejacket, the jet-ski type. Within 10 minutes, it had sunk,” he says.

**Figure:**
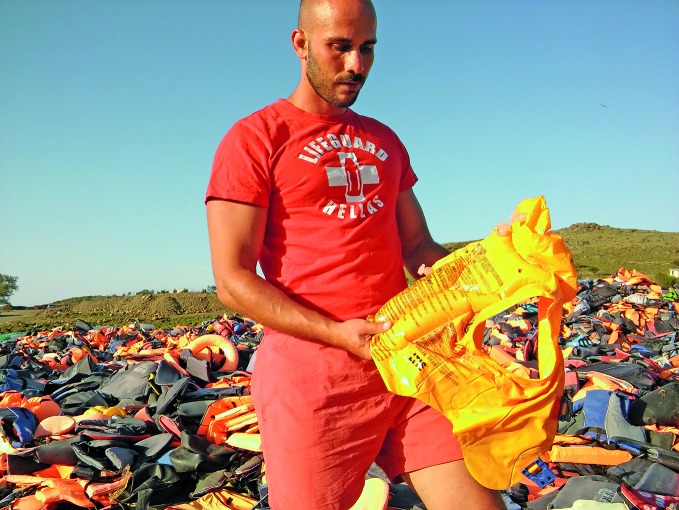
Volunteer lifeguard Nikolaos Mavreas at lifejacket cemetery, near Molyvos in northern Lesbos

Menelaos Tzafalias

A few weeks earlier, the decomposed body of a drowned woman, a refugee, had washed ashore at the same spot, Andreou says.

“We had drownings and we had a fake item. This wasn’t just about market fraud: it was about aiding and abetting murder,” says Andreou, who reported the incident to Save the Children International, one of the organizations responding to the refuge crisis.

“This wasn’t just about market fraud: it was about aiding and abetting murder.”

Nikolaos Andreou

Refugees have been crossing the Mediterranean Sea for years to escape political turmoil, conflict and poverty in the hope of a safer future in Europe.

But with deadly conflicts raging in the Syrian Arab Republic and in other parts of the Eastern Mediterranean, and in Africa and Asia, the number of people risking their lives on the hazardous sea crossing between Turkey and Greece has risen sharply.

Last year, according to the United Nations refugee agency (UNHCR), more than 500 000 people arrived on Lesbos and a further 89 000 arrived in the first three months of this year, having braved the crossing from Turkey, which can be as short as 7 km. 

“They come in rubber dinghies built for eight to 12 people, but with 60 or more on board. We’ve counted 88 people on a single vessel,” says Boris Cheshirkov, a spokesperson for UNHCR in Lesbos.

As the number of arrivals peaked last year, sometimes dozens of boats would land on Greek shores in a single day, he says. Since an agreement in March between Turkey and the European Union (EU), the arrivals have decreased but not stopped. 

Most of the refugees are smuggled into Greek waters by criminal gangs. The sea route is hazardous, the boats overcrowded and the death toll is high.

Last year, 3771 refugees – a record number – died attempting to cross the Mediterranean Sea (including the eastern Aegean). Most of them died crossing from Libya to Italy. About 20% (805) died crossing from Turkey to Greece, according the International Organization for Migration.

In the same year, criminal networks involved in smuggling refugees to and within the EU were estimated to have earned US$ 3.77 to 7.54 billion in their illicit trade, according to Europol.

Refugees drown when their overcrowded boats capsize or fill with water and sink. Without lifejackets made to international standards, the chances of survival are slim.

“The issue with illegal migration is that the vessels are not fit for purpose and have not been registered with any flag state,” says Ashok Mahapatra, Director of the Maritime Safety Division at the International Maritime Organization, the United Nations agency that regulates shipping.

The main requirements for a lifejacket are that it must be buoyant and stable enough in calm fresh water to be able to turn someone who is unconscious or face down in the water to a position where the mouth is clear of the water and incline the body backwards from the vertical position.

Not only must lifejacket manufacturers meet these requirements, but lifejackets must also be provided for every person on board a ship and each ship must be certified under the International Convention for the Safety of Life at Sea, Mahapatra notes.

**Figure:**
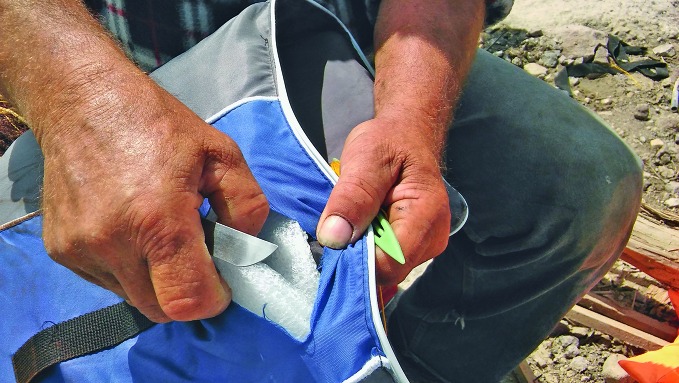
Fisherman on Lesbos cuts open fake lifejacket to reveal non-buoyant filling

Menelaos Tzafalias

Lifejacket cemetery – a makeshift dump for thousands of lifejackets in Lesbos – is a testament to the people who risked their lives crossing the Mediterranean.

There are inflatable vests for children with cartoon characters, life vests for water skiers and bright orange lifejacket fakes filled with foam and other materials that soak up water.

“The worst we’ve seen is compressed paper filling. We’ve even come across bubble wrap,” says Mania-Maria Bikof, who, with her husband, Spiros Mitritsakis, runs the Lifeguard Hellas Save and Rescue Volunteer team, the only such team from Greece operating in Lesbos.

“You’d see families with children wearing lifejackets and parents wearing blown-up inner tubes of car tyres,” she says, adding: “They had no idea that their lifejackets were fake and that inflated tyre tubes are safer.”

“We noticed that when people jumped off the boats, when reaching the shore, they would stumble,” Bikof says. “Later we realized they were weighed down by water-filled fake lifejackets.”

Bikof and her husband came to Lesbos a week after reading about the shipwreck in October 2015 off the coast of the island in which more than 70 people drowned.

Their lifeguard team is one of many volunteer groups that work in cooperation with the national Hellenic Coastguard and the EU’s border management agency, Frontex.

They ran a 24-hour lifeguard station on Limantziki beach with a team of 12 lifeguards until the last boat landed there in January, and have since been patrolling the sea every night in the Mytilini area.

As lifeguards they are trained and equipped to rescue people stranded at sea, but they had not seen anything like the situation off Lesbos before.

It is illegal to tow refugee boats with rescue vessels, to jump on board refugee boats or to take refugees on board rescue boats, unless the refugees’ boat is sinking, as such actions are considered to be human trafficking.

For every refugee boat that volunteer lifeguards want to help, rescuers must notify the Hellenic Coastguard. In emergencies, the Hellenic Coastguard allows the rescuers to take action to save the refugees.

“We helped more than 7500 people come in safely and disembark with volunteer lifeguards from 15 countries,” Bikof says, referring to dozens of qualified lifeguards who were referred to the Greek team by the International Surf Lifesaving Association. “During that time, we saw only one proper lifejacket.”

Some of the fake lifejackets come with CE (*Conformité Européenne*) and ISO (International Organization for Standardization) markings to dupe desperate refugees into thinking they are manufactured according to international standards.

The Turkish authorities have been investigating the illegal manufacture and trade in these lifejackets at least since last year, according to media reports.

Turkish police raided a workshop in the coastal city of Izmir in January, where fake lifejackets were being manufactured, seized over 1200 jackets filled with non-buoyant material and made several arrests.

The raid came a day after the bodies of several refugees had washed ashore on Turkish beaches, some of them were wearing lifejackets, following two shipwrecks, according to media reports.

“Unless you can swim ashore after an incident at sea, the prospect of survival comes down to whether you can keep your head above water long enough for a rescue vessel to find you,” says Dr David Meddings, a drowning prevention expert from the World Health Organization’s Department for the Management of Noncommunicable Diseases, Disability, Violence and Injury Prevention.

“That’s where lifejackets come in. The term ‘lifejacket’ refers to something designed for use on the open sea, where immediate rescue is unlikely.

“They are different to other personal flotation devices – sometimes called life vests – that have less buoyancy and will not routinely turn an unconscious person face up as they are designed for use in recreational settings where immediate rescue is likely,” Meddings says.

“Each death from drowning is a tragedy and a tragedy that – in many cases – can be averted.”

David Meddings

“Refugee drownings are a stark reminder of the risks inherent in transport over water. All countries can reduce these risks by setting and enforcing regulations for safe boating, shipping and ferry transport, including regulations regarding appropriate and certified personal flotation devices.

“Each death from drowning is a tragedy and a tragedy that – in many cases – can be averted,” says Meddings.

The rescuers from Greece and other countries share that sentiment. The Lifeguard Hellas team keeps watch all night, sleeping at the beach and maintaining rescue boats ready to respond. Many team members who joined the initial response last year, like Nikolaos Mavreas, have returned again and again, volunteering any free time they have between work and family commitments.

“I felt that since this was happening in my country I had to help. If something similar happened to me, I would appreciate it if someone helped,” says Mavreas, a personal trainer and a former captain of the Greek national rugby team.

